# Overfat Adults and Children in Developed Countries: The Public Health Importance of Identifying Excess Body Fat

**DOI:** 10.3389/fpubh.2017.00190

**Published:** 2017-07-24

**Authors:** Philip B. Maffetone, Ivan Rivera-Dominguez, Paul B. Laursen

**Affiliations:** ^1^Independent Researcher, Oracle, AZ, United States; ^2^Research Assistant, San Diego, CA, United States; ^3^Sports Performance Research Institute New Zealand (SPRINZ), Auckland University of Technology, Auckland, New Zealand

**Keywords:** obesity, abdominal obesity, overweight, chronic disease, inflammation, insulin resistance

## Abstract

The global overfat pandemic is a serious public health crisis that places a substantial burden on economic resources in developed countries. The term *overfat* refers to the presence of excess body fat that can impair health, even for normal weight non-obese individuals. Excess body fat is associated with cardiometabolic dysfunction, a clinical situation that can progressively worsen, potentially leading to various common disease risk factors, chronic diseases, increased morbidity and mortality, and reduced quality of life. The prevalence of overfat populations in 30 of the world’s most developed countries is substantially higher than recent global estimations, with the largest growth due to a relatively recent increased number of people with excess abdominal fat. Abdominal overfat is the most unhealthful form of this condition, so it is concerning that average waist circumference measures, generally indicative of abdominal overfat, have increased. Despite a leveling off appearance of being overweight and/or obese in some developed countries, the overfat pandemic continues to grow.

## Key Findings

On average, the prevalence of overfat adults and children in developed countries is extremely high, and substantially greater than that of overweight and obese individuals.
In the US, New Zealand, Greece, and Iceland, prevalence of the overfat condition is at an alarmingly high rate of over 90% in adult males and up to 50% in children.
Despite a leveling off appearance of the overweight and/or obese condition in some developed countries, the overfat pandemic continues to grow.
In tandem with an increased average waist circumference, a recent rise in the incidence of abdominal adiposity, the unhealthiest form of excess body fat, has been observed in both adults and children.

## Introduction

It was recently estimated that between 62 and 76% of the world’s population have reached body fat levels that can impair health (1). This condition, which can now be labeled a pandemic, was described by the catch-all term *overfat* (Table 1). It is well-recognized that the overweight and obese conditions represent a continuing threat to world health, replacing more traditional problems of undernutrition and infectious diseases. Indeed, being overfat shares direct links to insulin resistance and chronic inflammation, and to hypertension, dyslipidemia, coronary heart disease, stroke, cancer, Type 2 diabetes, gallbladder disease, osteoarthritis and gout, pulmonary diseases, sleep apnea, and others (2). Global rates of these conditions in adults and children (including adolescents) have risen significantly over the past ~40 years, paralleling significant increases in the numbers classified as being overweight and obese, and considerably affecting people of all ages and incomes in both developed and developing countries (3).

**Table 1:** Commonly used abbreviations and definitions.

Term	Abbreviation	Definition
Overfat	–	Excess body fat that can impair health
Body fat percentage	BFP	Total% body fat vs. lean mass
Body mass index	BMI	Weight in kilograms divided by height in square meters
Metabolically obese normal weight	MONW	Normal-weight overfat with cardiometabolic dysfunction
Normal weight obese	NWO	Normal weight overfat with cardiometabolic dysfunction
Metabolically healthy obese	MHO	Overfat with little/no measurable health risk
Waist circumference	WC	Measure of waist in centimeters
Waist-to-height ratio	WHtR	Ratio of waist-to-height in centimeters

While the prevalence of being overweight and obese is well known (4), many normal-weight and non-obese individuals exhibit excess levels of body fat that can adversely affect their health (5–7). Indeed, reliance of body mass index (BMI) for determination of being overweight and obese may misclassify up to 50% or more of patients with excess body fat who may have increased health risks (8, 9). The notion of a metabolically obese normal weight (MONW) individual is based on the finding that obesity-associated disorders such as high circulating insulin levels in people with cardiovascular disease or Type 2 diabetes can occur in those with normal BMI (10–14). Many at-risk individuals have been identified in a BMI range of 23–25 or lower (11). Overfat individuals who are not overweight and obese include MONW individuals (10, 15, 16), those with sarcopenic obesity (17), and many who have increased abdominal fat stores. Abdominal and visceral fat accumulation, regardless of weight status, has been found to increase risk of cardiovascular and metabolic (cardiometabolic) disease to the greatest degree (10, 18–20).

In addition, the similar concept of *normal weight obesity* (NWO) expands on the notion of MONW by describing the association between normal weight and high body fat percentage (BFP) with cardiometabolic abnormalities (21). Oliveros et al. (22) refer to MONW individuals as a likely subset of NWO people. Thus, we include both categories in our estimations of overfat populations herein.

Body mass index is calculated as body weight (in kilograms) divided by height (in meters) squared, with the World Health Organization (WHO) defining overweight as having a BMI ≥ 25–29.9 and obesity as having a BMI ≥ 30 kg/m^2^ (2, 4). While it is well-known that central to the pathophysiology of obesity is an excess amount of adiposity, clinicians and researchers usually rely on BMI (which does not directly estimate body fat) to define the presence of adiposity or obesity—a practice strengthened by the discovery of an association between BMI and increased mortality (23).

However, large multiethnic samples from the US general population have demonstrated that BMI has limited diagnostic performance in correctly identifying individuals with excess body fat, particularly in those with BMI < 30 kg/m^2^, with BMI missing more than half of people with BFP-defined obesity (8, 9). For example, Hung has shown that up to 70% of young women with high BFP would be missed by BMI category alone (24). The main limitation of BMI is that it cannot differentiate fat mass from lean mass, or central from peripheral fat (5, 22). Furthermore, the relationship between BMI and BFP varies considerably among different ethnic groups, further lowering the utility of BMI as a predictive measure of adiposity and health risk for many groups of people (25, 26).

Regardless of body weight or BMI, excess BFP in adults is associated with a wide range of cardiometabolic dysregulation, as indicated by various risk factors associated with downstream disease and mortality (5–7). In particular, excess BFP is associated with elevated fasting triglycerides and glucose, low HDL and high LDL cholesterol, increased blood pressure, a higher odds ratio of developing the metabolic syndrome, along with Type 2 diabetes, cardiovascular disease, and other chronic illness, with increased mortality as a consequence. The earliest dysregulation is often observed now in children (10, 27).

**Figure 1:**
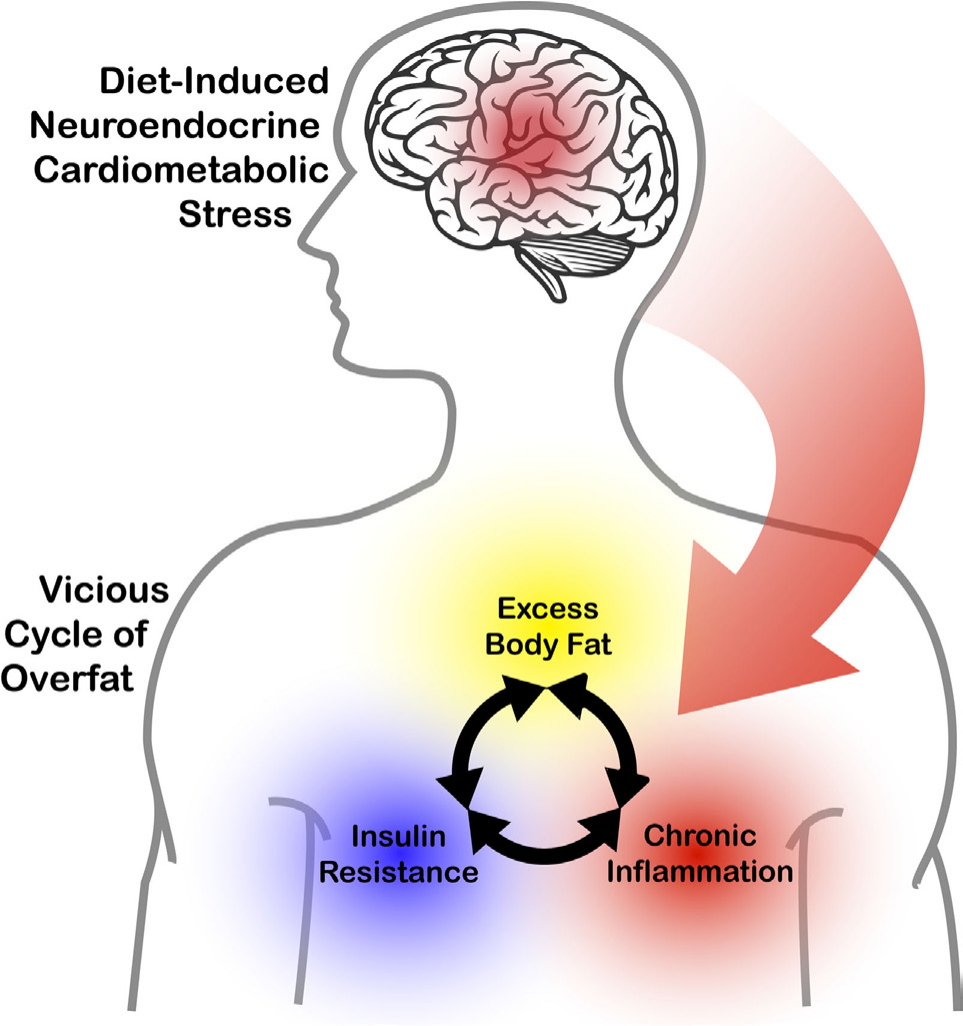
The impact of diet-induced neuroendocrine cardiometabolic stress on the interrelationship between excess body fat, chronic inflammation, and insulin resistance.

Perhaps the earliest onset of cardiometabolic dysregulation begins as a vicious cycle of excess body fat, insulin resistance, and chronic, low-grade systemic inflammation (28, 29). Excess adiposity is a source of inflammatory molecules that can disrupt glucose homeostasis and contribute to the pathogenesis of insulin resistance (30, 31). In turn, insulin resistance further exacerbates adiposity by influencing glucose disposal and fat storage. As such, the overfat condition potentially contributes to two adverse metabolic conditions (chronic inflammation and insulin resistance), which then contribute to further adiposity. This is in effect a vicious cycle wherein the appearance of adiposity and its initial consequences feed back into themselves, accelerating and entrenching the dysregulation (See Figure 1).

An updated definition of obesity based on adiposity, not on body weight, is urgently needed (22). As we have argued, the term overfat fills this void. As such, the term may be better suited to help public health efforts in reducing cardiometabolic risk factors and preventing disease, improving quality of life, and mitigating the economic burden of this global problem.

### Calculation of Fat Mass

The methods used to quantify BFP include bioelectrical impedance, hydrostatic plethysmography, isotope dilution techniques, dual x-ray absorptiometry (DXA), skinfold method, body impedance measures, and others, with DXA one of the most accurate and precise methods available to measure total body fat and lean soft tissue mass directly (32). Epidemiologic studies have also demonstrated that central fat distribution assessed through waist circumference (WC), waist-to-hip ratio, and waist-to-height ratio (WHtR) measures can be very useful in assessing adiposity-related risk (22); although, like BMI, these measurements cannot estimate BFP.

While there is still no consensus on normal BFP cutoffs, Gallagher et al. (33) suggest they should vary with age such as 20–39 years, >19 and >32%; 40–59 years, >21 and 33%; and 60–79 years, >24 and 35% for men and women, respectively (33). Body fat distribution, genetics, and fitness also can contribute to the development of health risk factors.

Recent large-scale epidemiological analyses, such as the 2013 Global Burden of Disease (GBD) study reported in Ng et al. (4), have attempted to provide a picture of obesity-related health risk on a worldwide scale, defining the prevalence of overweight and obese conditions using common BMI conventions for adults (overweight as ≥25 to <30 kg/m^2^ and obesity as ≥30 kg/m^2^). Childhood obesity is often based on the International Obesity Task Force definition, which established definitions for global classification of children as overweight and obese (34).

The implications of relying on BMI as a principal measure of obesity are not limited to the context of clinical diagnosis of individuals. Indeed, doctors often have access to other tools that allow them to correct for the drawbacks of BMI. The larger problem is that most large-scale epidemiological studies that use BMI have no way of correcting for the metric’s disadvantages. As a result, these studies may have substantially underreported the population of individuals who are at risk of obesity-related cardiometabolic disease. Effectively, overreliance on BMI may have serious implications on our ability to size the problem, and therefore on our ability to properly address its consequences.

A central purpose of this paper is to take recent theory and data on normal weight non-obese populations at cardiometabolic risk due to excess body fat, combine them with those who are overweight and obese, and provide preliminary estimates of overfat people in 30 of the most developed countries according to the Human Development Index (HDI), as described in the Human Development Report for 2014, authored by the United Nations Development Program (35). Our expectation was that developed countries would show a higher proportion of overfat individuals, in part due to lower levels of underfat, with developing nations outnumbering developed ones by more than 6:1 (36).

## Overfat as a Risk Factor

Overfat individuals may be at increased risk for developing a wide range of health problems, including cardiometabolic abnormalities, downstream diseases, and increased morbidity and mortality (5, 37). Overweight and obesity alone represents the fifth leading risk for global deaths (38) and is projected to become the most common modifiable factor among preventable causes of global death (39). Estimating the extensive prevalence of overfat can add to our public health knowledge in addressing the pandemic, including preventing the earliest forms of chronic illness.

### Clustering of Subclinical Risk Factors

The potential for a single risk factor to have clinical value should not be underestimated and could very well be the earliest sign of future disease (40). The overfat condition can be understood as a subclinical risk factor, which can precede the more significant cardiometabolic dysregulations noted above. Increased WC alone, such as in normal-weight individuals, is associated with the metabolic syndrome, as are other subclinical conditions such as prediabetes (41) and prehypertension (42).

The National Health and Nutrition Examination Survey (NHANES) III attempted to establish the relationship between health and nutrition in the United States population. In this study, within the group of normal-weight adults with higher BFP, the prevalence of metabolic syndrome and its individual components was shown to increase linearly with increases in BFP (5). In a study of Brazilian adults (20–23 years), higher BFP in normal weight was also associated with metabolic syndrome and insulin resistance (43).

The clustering of risk factors, such as those observed in metabolic syndrome, MONW, and NWO, suggests that more than one primary abnormality typically exists in certain individuals. For example, a predisposition to glucose intolerance, associated with insulin resistance, may be a common factor in the case of those who are overfat (44). A significant number of other cardiometabolic risk factors were also shown in up to 30% of normal weight, non-obese male individuals who demonstrated impaired fasting glucose (45). Adiposity and weight gain were key determinants of the degree of clustering of cardiometabolic risk factors in Framingham study participants, with increased WC being a primary factor (40). Within this larger clustering occurs the chronic, smaller category of metabolic syndrome. Wilson and Meigs (40) state that, “Excess adiposity, especially abdominal adiposity, and insulin resistance are probably key ingredients that lead to the development of the risk factor abnormalities that then translate into the clinical outcomes.”

Unfortunately, most of the studies demonstrating clustering of common conditions do not include well-described subclinical disorders that can pre-date disease states, such as prehypertension and prediabetes. Future studies should address relationships between these very early risk factors with that of glucose intolerance and insulin resistance, and its impact on cardiometabolic health (46, 47). This might also include evaluating overfat children, whose conditions can play a key role in the advent of downstream diseases typically exhibited in adulthood. Unfortunately, adiposity in children is likely to feed the overfat pandemic because research shows that obese children are at increased risk for becoming obese adults with chronic disease (48–53).

### Clustering of Clinical Risk Factors

**Figure 2:**
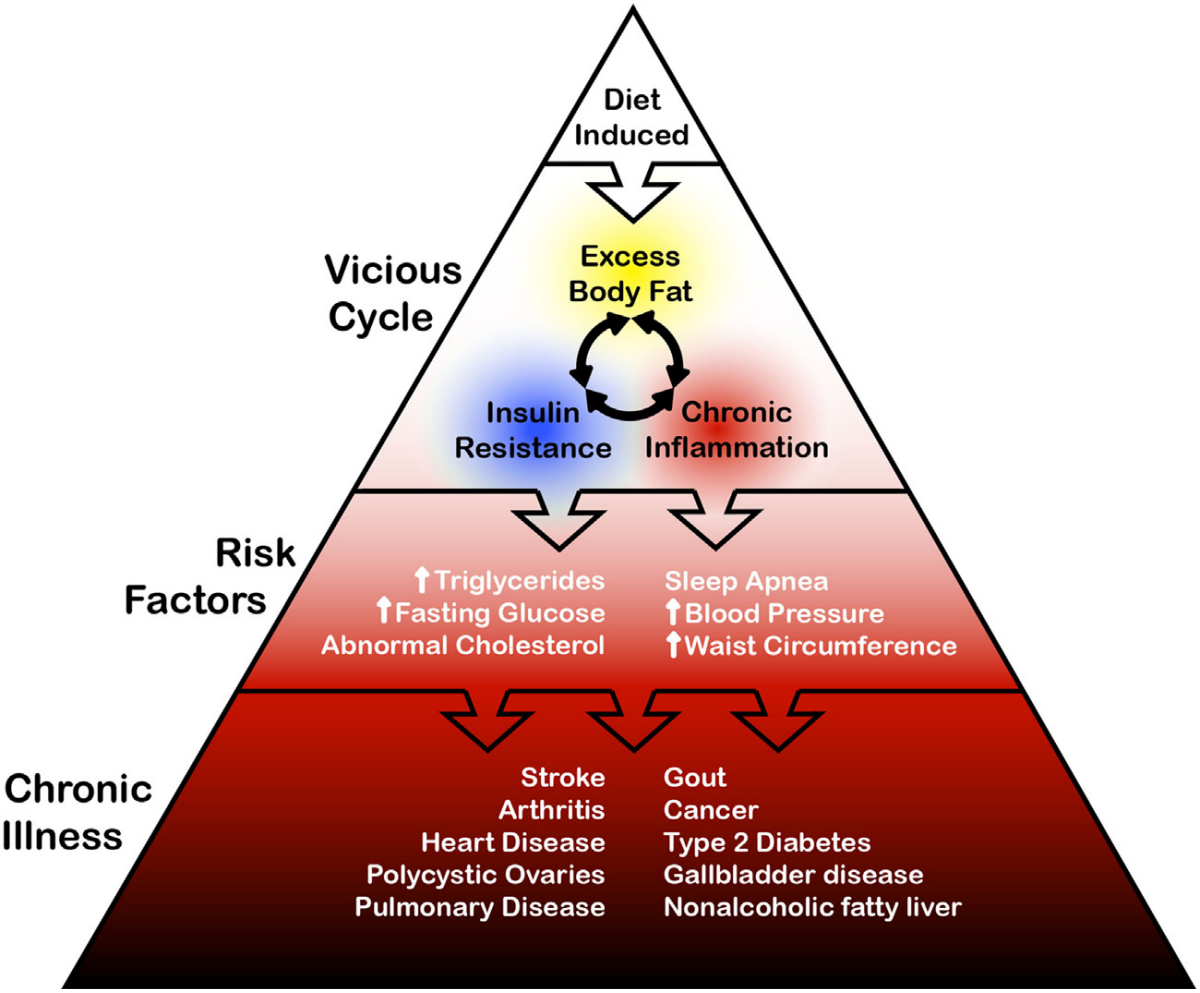
General schematic of relationships between diet, overfat, some risk factors, and chronic illness.

Since the early observations of Ruderman et al. in 1981, the list of associated disorders clustered in normal-weight overfat populations has grown (11). Rather than thinking of these disorders as separate, individual clinical problems, it might be best to consider the relationships between overfat, its various risks, and its associated downstream diseases as a spectrum or progression where the vicious cycle of overfat, insulin resistance and chronic inflammation lies at one end, (constituting the population with early measurable abnormality), while the presence of chronic conditions resides at the other end (see Figure 2).

Prevalence of the conditions listed in Figure 2 is also increased in individuals with a history of low birth weight, as well as in normal-weight (<25 BMI) offspring and other first-degree relatives of patients with these conditions (10). A preliminary evaluation of the offspring (mean age 54 years) of the original Framingham study cohort found that 44% of the population had one or more abnormal findings associated with insulin resistance (54). Other associated disorders include (1) neuroinflammation, which can affect the function of the hippocampus, cortex, brainstem, or amygdala, with clinical conditions such as depression and impaired cognitive function (55), as well as (2) reduced quality of life, which includes a twofold risk of physical activity difficulties such as walking and climbing stairs, increased pain, and hindrance to daily activities (56).

Another cardiometabolic risk is cancer-related clustering of prediabetes, diabetes, metabolic syndrome, and chronic inflammation (57). The cancer-obesity connection in the past was limited to a small number of cancers. However, Lauby-Secretan et al. (58) recently emphasized that the absence of excess body fat lowers the risk of most cancers and discussed the possibility that the association between higher body fat and cancer risk is causal. In addition, survival rates among persons with chronic diseases such as cancer may decrease, as increased body fat is associated with worse treatment outcomes (59).

To summarize, whether an individual presently meets the criteria for NWO but not MONW (or for MONW but not for metabolic syndrome) is inconsequential to whether they are overfat, and therefore at increased risk of downstream cardiometabolic impairment.

### Is Obesity Leveling? Overfat Is Not

Based on BMI evaluations, there appears to be a leveling off of the trend in rising obesity rates in some developed nations (60, 61). However, the incidence of central adiposity—the excess accumulation of visceral fat in the abdominal region, sometimes called abdominal obesity (62)—is increasing (61). This form of overfat is concerning because the potential health risks of central adiposity are more pronounced than those for increased subcutaneous fat in other regions of the body (63). The continued increase in abdominal obesity includes those who are normal weight and non-obese, with US population averages of 54.2% (51.3–57%), and an increased prevalence in women (up to 68.3%) (61).

It may be too early to make the claim on a leveling of obesity rates for various reasons. Previous stable phases of the obesity epidemic have been followed by further increases (64). There are also increased rates of abdominal obesity as indicated by increases in WC (61, 65). While obesity has recently plateaued at 35% for US men, it continues to increase to 40% for women, with a similar trend in the 60 years and older group (66), with morbid obesity rates continuing to rise in developed countries (67, 68). One billion adults are projected to have obesity by 2025, far more than the current levels (69). In addition, younger generations are becoming obese at an earlier age and are staying obese for a longer duration than older generations (70).

The notion that obesity in some countries has slowed or leveled may constitute an inconsistent public health message that erroneously implies we are solving a serious problem while the overfat pandemic continues to grow. Despite the trends in overfat, weight counseling and clinical diagnosis of overweight and obesity in US primary care significantly declined between 1995–1996 and 2007–2008 (71, 72). In addition, fewer overweight and obese adults report trying to lose weight between 1988–1994 and 2009–2014, with percentages declining from 56 to 49%, respectively (73).

### Metabolically Healthy Obese (MHO)

Researchers have described MHO individuals, a prevalence estimated at 7.27% (74), who, despite having BMI > 30, appear relatively insulin sensitive and lack some or most of the metabolic abnormalities typical of obese individuals (75–78). Even though conditions such as metabolic syndrome may not be prevalent in MHO individuals, the presence of fewer (or other) cardiometabolic risk factors typically associated with obesity can still exist. Within these, being overfat is the critical factor that confers risk for Type 2 diabetes and CVD associated with adiposity (37). In a large, community-based sample of men and women across a broad age spectrum, Meigs et al. (37) stated that MHO subjects were younger than obese subjects with metabolic syndrome, but as they age, they may transition from obese and apparently healthy to obese with risk factor clustering. This implies that MHO individuals may be in the process of becoming metabolically unhealthy. Wang et al. (74) showed that the prevalence of MHO was lower for adults > 40 years than the general population, and that these individuals were at increased risk for all-cause mortality and/or cardiovascular events over the long-term (≥10 years). Other studies have noted that these seemingly healthy obese subjects may have subclinical disease, concluding that longer follow-ups or more careful evaluation may be required before referring to them as healthy (79, 80). As such, we consider so-called MHO individuals to also be overfat (22).

### Economics of Overfat

The economic fallout from the overfat pandemic has raised a serious global challenge. In 2011, the WHO estimated that the economic burden of preventable, non-communicable disease (in particular cardiovascular disease, cancer, and diabetes) is expected to create a cumulative output loss of US$47 trillion over the next two decades (81). In 2010, this represented 75% of global GDP (US$ 63 trillion)—enough capital to lift the 2.5 billion people currently below the poverty line, out of poverty for more than half a century. While it is difficult to determine the absolute burden of the overfat pandemic, it is clearly a strong causal factor in the development of a significant portion of chronic disease and reduced quality of life.

## Who is Overfat

It is important to recognize high-risk populations for better adiposity-based risk stratification, along with a need for an updated definition of obesity based on adiposity, not on body weight (22). We previously estimated the global prevalence of the overfat population using several subpopulations that included those who are overweight, obese, MONW individuals, those with sarcopenic obesity and others (1). Additionally, the overfat pandemic has not spared physically active people, including professional athletes in various sports (82, 83) and active US military personnel (84, 85).

Unique to developed countries are the recently increased numbers of older persons more likely to develop sarcopenic obesity, and the increase in abdominal obesity that outpaces increases in BMI. We introduce two new additional terms to more accurately describe excess fat accumulation in those who are sarcopenic and those with increased abdominal adiposity. Specifically, because the term “obesity” has historically been explicitly based on BMI, its use to describe these two body compositions is arguably incorrect and may even be confusing to the public and media. We therefore aim to better define and draw attention to two growing components of the overfat pandemic, sarcopenic overfat, and abdominal overfat.

### Sarcopenic Overfat

Sarcopenia is defined as the progressive loss of type II fast-twitch muscle fibers and strength with aging and is an important public health problem with a prevalence as high as 50% in those >80 years (86). Muscle atrophy can coexist with an accumulation of fat within existing muscle, and the combination of higher body fat and sarcopenia has been termed sarcopenic obesity (87, 88), which we refer to henceforth in this paper as *sarcopenic overfat*. This condition is associated with low-grade chronic inflammation, insulin resistance, elevated C-reactive protein, and excessive oxidative stress (17). Sarcopenia increases the risk of disability and poor quality of life, and death, and may have an important association with chronic illness and aging. In the elderly, who naturally exhibit the highest rates of mortality in survival studies, the diagnostic performance of BMI is at its worst, likely to be highly under-estimating the extent of overfat people with sarcopenic overfat (8).

### Abdominal Overfat

The increasing rates of overfat people are due in great part to increased incidence of abdominal obesity in adults and children (61, 89). Other terminologies used for this condition include visceral obesity, visceral adiposity, android obesity, central obesity, the hypertriglyceridemic waist, and others. Since the problem can occur in people who are not obese, we suggest the term *abdominal overfat* as a simplified one that broadly defines the specific problem of excess abdominal fat.

Two separate components of abdominal fat include visceral adipose tissue and subcutaneous adipose tissue, the former being associated with more adverse risk factor profiles than the latter (45, 90). Although total adiposity is strongly associated with cardiometabolic risk, it is becoming increasingly clear that visceral adipose tissue is a stronger correlate of risk than BMI, WC, or abdominal subcutaneous fat (91), and more strongly associated with insulin resistance, dyslipidemia, and atherosclerosis than peripheral obesity (92).

The recognition that abdominal overfat has separate and more severe health effects is not new (93–106). Prospective epidemiological studies have demonstrated the power of abdominal overfat to predict premature death in conjunction with various cardiometabolic-related chronic diseases in both men and women (107). Numerous studies more recently reported secular increases in WC in men and women in the US, England, Australia, Finland, and the Netherlands (108–115).

In our estimation of overfat children, we considered studies that measured BFP and WHtR. BFP measures may represent a reasonable method of estimating the population of normal-weight non-obese children, as excess body fat can exist in those with a BMI < 25 (116). Based on the WHtR (<0.5 vs. ≥0.5), 37% of normal-weight non-obese children had at least one cardiometabolic health risk factor (associated with WC, triglycerides, and high blood pressure), 13.5% had at least two risk factors, and 0.3% had three risk factors (117). Moreover, as with adults, abdominal overfat is associated with greater health risks in children (118, 119).

**Figure 3:**
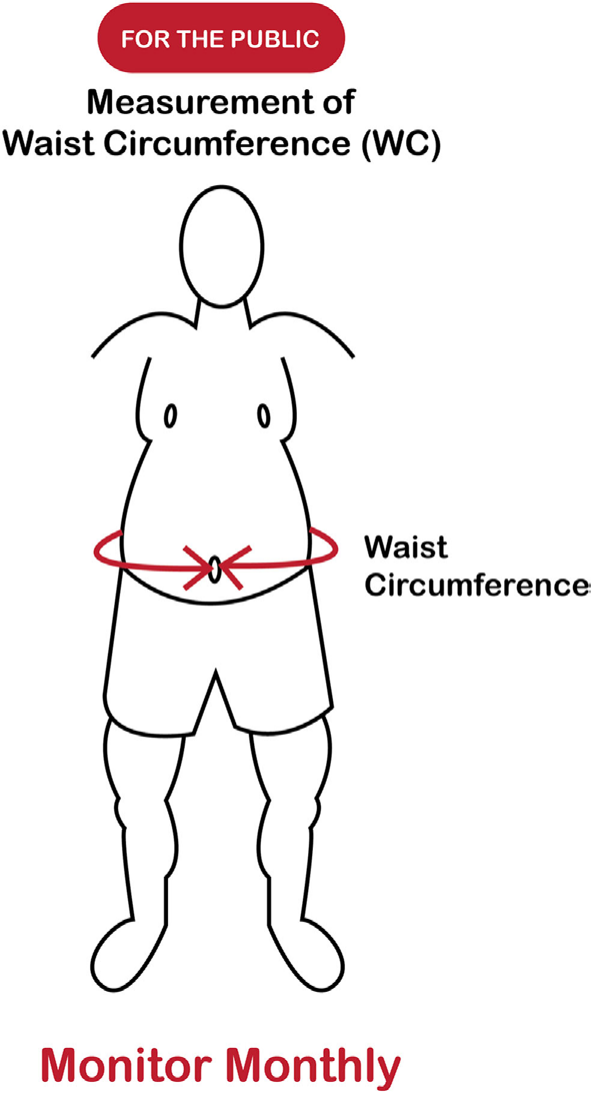
Measuring overfat (for the public).

**Figure 4:**
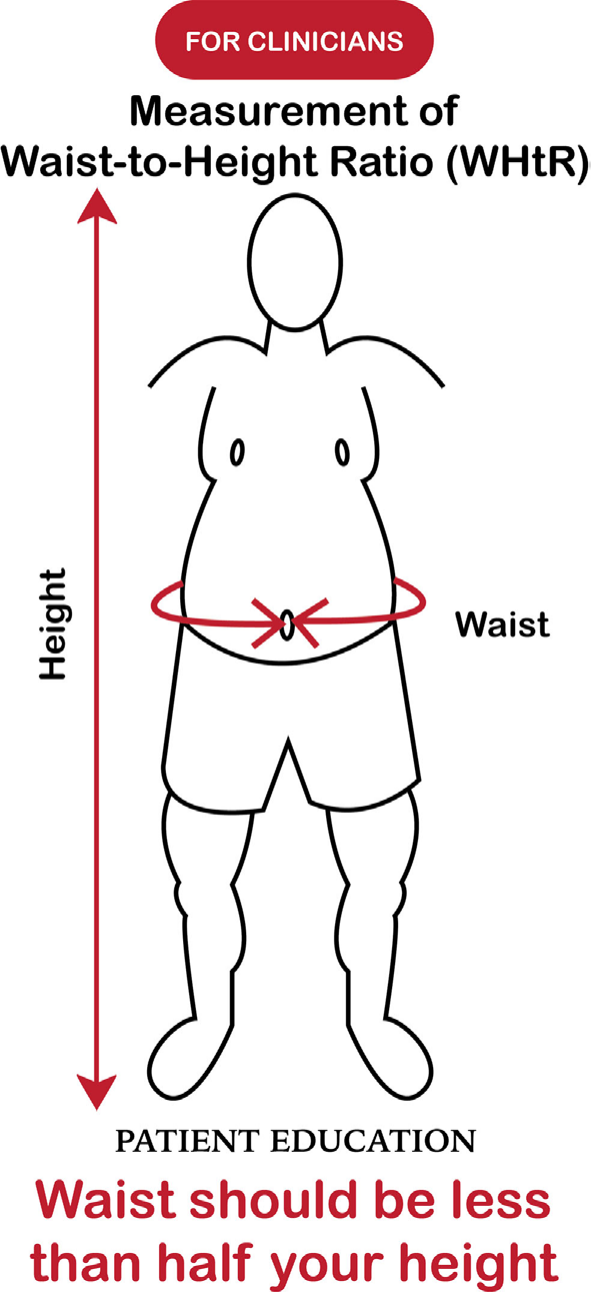
Measuring overfat (for healthcare practitioners).

In recent decades, the prevalence of childhood abdominal overfat has increased more quickly than the prevalence of obesity as defined by BMI (120, 121). While, as in adults, research shows an unexpected plateauing of childhood obesity rates in some developed countries (122), increased rates of abdominal overfat appear to have maintained the overall growth rate of the overfat pandemic. In addition, extreme obesity in children continues to increase despite the fact that rates for lower obesity categories are leveling or even declining (123). In light of the clear limitations of the BMI described herein, the WHtR may be the single best clinical indicator of health risk as it can be used throughout childhood, into adult life, as well as throughout the world (in all ethnic groups). (See Figures 3 and 4) (124, 125).

Additionally, as an important part of clinical practice, the routine measurement of WC is consistent with current recommendations for the prevention, control, and management of overfat in patients (126).

## Estimating Overfat Populations

**Figure 5:**
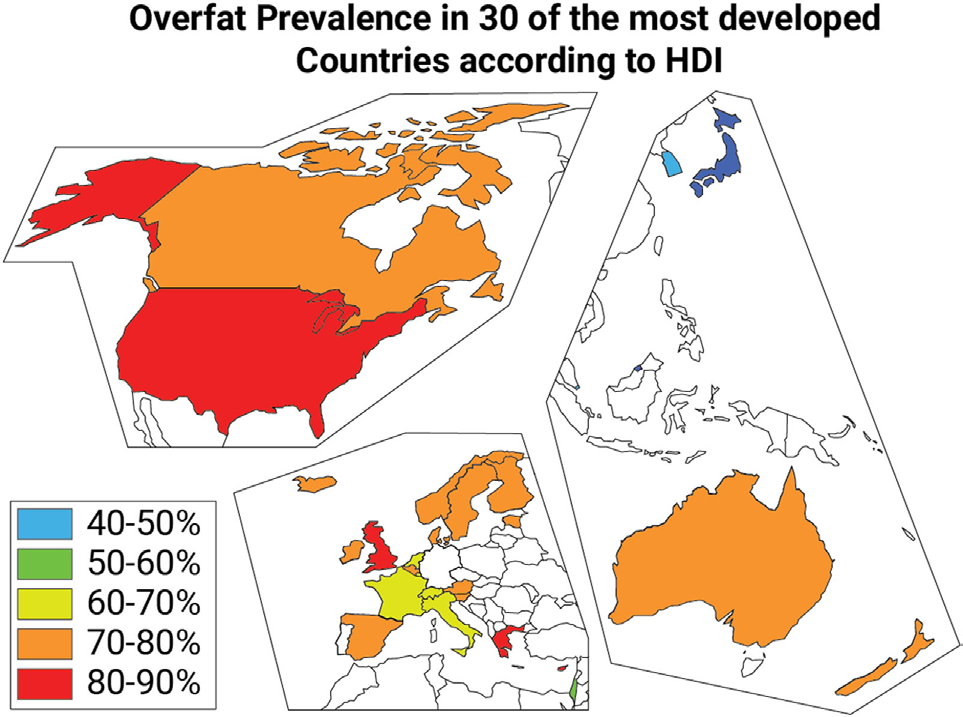
Overfat prevalence categories in 30 of the most developed countries according to the Human Development Index.

We estimated the incidence of overfat in 30 developed nations listed in Table 2 using the HDI described by the United Nations Development Programme’s Human Development Report for 2014 (see Figure 5). Accurate estimations of the prevalence of overfat populations is challenging, especially considering that appropriate BFP analyses (e.g., X-ray absorptiometry, DXA) are not common. However, a number of studies have been helpful in differentiating normal-weight and non-obese but overfat individuals from those who are overweight or obese (8, 9, 127) As such, in addition to the estimations of overweight and obese populations (4), we also considered individuals with increased BFP and or WC/WHtR in normal-weight non-obese adults with signs of cardiometabolic impairments as discussed above, including two national studies in the UK and US, which provided useful data on children who were normal weight and not obese. Griffiths et al. (128) showed that 17.6% of normal-weight non-obese boys and 17.3% of normal-weight non-obese girls who exceeded a WHtR of 0.5 were considered “at risk,” as compared with children who were overweight or obese and above the 85th percentile (128). Flegal et al. (116) estimated the prevalence of high adiposity for children below the 85th percentile using dual-energy X-ray absorptiometry to estimate BFP and found that a high number of children with normal BMI had high adiposity—35% of children within the 65th percentile cutoff and 24% of children within the 70th percentile cutoff.

**Table 2:** Percentages of overfat subpopulations of the 30 most developed countries according to the Human Development Index.

	Overfat (%)			

Country	Adults		Children	

	Males	Females	Males	Females
Norway	78.4 (75.7–81)	67.3 (64.4–70.2)	42.7 (39.8–45.6)	38.2 (35.6–40.9)
Australia	88.2 (85.6–90.5)	76.1 (73.4–78.9)	47 (44–50.6)	45.2 (42.1–48.7)
Switzerland	76.6 (73.7–79.4)	59.9 (57–62.9)	43.3 (40–47)	38.4 (35.6–41.6)
Denmark	79.2 (76.5–81.9)	64.7 (61.7–67.7)	42.3 (39.4–45.7)	41.6 (38–45.4)
Netherlands	73.2 (71.1–75.4)	64.9 (62.3–67.5)	40.9 (38.3–43.9)	38.3 (35.6–41.1)
Germany	84.3 (81.9–86.8)	69 (66.5–71.4)	43.1 (40–46.4)	41.6 (38.5–44.7)
Ireland	86.4 (83.9–88.8)	70.9 (68.3–73.6)	49.2 (45.8–53.4)	48.7 (45.1–52.7)
United States	90.9 (89.2–92.5)	81.9 (79.8–83.8)	51.4 (49–54)	51.9 (49.4–54.7)
Canada	84.5 (82–87)	68.5 (65.9–71.1)	48.1 (45–51.3)	44.2 (41.3–47.7)
New Zealand	91.4 (89.6–93.3)	80 (77.8–82.2)	52.2 (48.6–55.9)	50.9 (47.5–54.8)
Singapore	64.3 (61.4–67.1)	52.5 (50–55.1)	43.5 (40.1–46.9)	35.5 (33.1–38.2)
Sweden	78.2 (75.6–81)	65.8 (63.2–68.5)	43 (40.1–46)	41.5 (38.7–44.7)
United Kingdom	86.6 (85.3–88)	77.2 (75.7–78.6)	48.7 (46.4–51.1)	51.4 (49–54.1)
Iceland	93.6 (91.3–95.8)	80.9 (78–83.8)	49 (45.3–52.8)	45.2 (41.9–48.8)
South Korea	56.9 (55.1–58.8)	47.2 (45.6–48.9)	43.8 (40.5–47.1)	35.4 (33.1–37.9)
Israel	80.4 (77.6–83.2)	72.7 (69.6–75.6)	53.6 (49.6–58.2)	48.8 (44.8–53.3)
Luxembourg	78 (75.1–80.8)	64.4 (61.6–67.2)	51.9 (47.9–56)	39.9 (36.7–43.3)
Japan	48.9 (47.1–50.7)	37.6 (36.5–38.9)	37.9 (35.8–40.2)	34.6 (32.4–36.8)
Belgium	78 (75.2–80.8)	67.1 (64.3–69.9)	43.1 (40.3–46.2)	41 (38.2–44)
France	75.9 (73.2–78.7)	62.8 (60–65.7)	42.5 (39.4–45.9)	38.2 (35.5–40.9)
Austria	79.7 (77–82.3)	62.8 (60.1–65.4)	41.5 (38.5–44.7)	38.5 (35.7–41.6)
Finland	82.2 (79.5–84.9)	70.4 (67.5–73.2)	48.6 (44.9–52.4)	43.3 (39.9–47.2)
Slovenia	85.1 (82.3–87.6)	72.1 (69.1–74.8)	55.7 (52–59.5)	46.2 (42.9–49.5)
Spain	82.3 (80–84.9)	66.5 (63.7–68.9)	50.2 (46.5–53.8)	46 (42.4–49.6)
Italy	78.3 (75.5–81.1)	61.4 (58.9–64.2)	52.5 (49–56.5)	46.5 (43.2–50.1)
Czech Republic	85.5 (82.9–88.2)	70 (67.2–72.7)	44.9 (41.7–48.9)	40.2 (37.2–43.2)
Greece	91.4 (88.9–93.7)	71.1 (68.2–74)	56.3 (52.2–60.3)	51.3 (47.5–55.3)
Estonia	79.3 (76.5–82)	74.3 (71.5–77.2)	46.6 (42.8–50.4)	43.6 (40.2–47.4)
Brunei	43.3 (41.2–45.2)	37.9 (36.2–39.8)	29.3 (28.1–30.6)	27.8 (26.7–29)
Cyprus	87.8 (85–90.6)	72.1 (69.1–75.1)	48.3 (44.5–52.2)	44.7 (41.1–48.4)

Our estimates did not include normal-weight non-obese people who had high fat mass but only early signs of cardiometabolic dysfunction (such as prediabetes or prehypertension), because insufficient data were available. However, the numbers of individuals in this category could be significant. For example, prediabetes in the US has risen to ~34% in the population even among those who are normal weight (129). Prehypertension is also not uncommon and increases the risk for incident hypertension and cardiovascular mortality (130).

Since data for overfat subpopulations was not uniformly available for the same year, we used the most recent data available in order to produce our estimates. Ng et al. (4) used a similar strategy to provide estimates of various populations at risk of metabolic disease, as do other large-scale epidemiological studies cited throughout our paper. The most recent data, obtained from the Centers for Disease Control and Prevention (CDC) website shows that 71% of US adults were overweight or obese (131). However, for continuity, our analysis for all countries used data from Ng et al. (4), which shows overweight and obesity in US adults at 66%. Had we used the more recent CDC data, our estimate for the US would likely have yielded much higher percentages of overfat.

In our estimation of overfat adults and children, it was not possible to precisely quantify all categories of overfat individuals. One limitation of our estimates is that we may have underrated those 70–80 years and older with sarcopenic overfat, and those who were institutionalized or otherwise in poor health, as these individuals are usually not part of community and other population studies. In addition, younger children up to 5 years are often not counted in population studies. Children between the ages of 5 and 14 years may have already been overfat when entering kindergarten (132). The use of WC and WHtR often use relatively high cutoff points and may miss early conditions of overfat potentially making our estimates conservative. Likewise, BFP in most adult studies typically use relatively high cutoff points recommended by the WHO of 30 for men and 35 for women, furthering the risk of underreporting overfat individuals.

The importance of our review includes increasing awareness of the overfat pandemic in developed countries, whose rates are relatively higher than worldwide percentages. In addition, we have emphasized the continued increase of overfat populations despite the appearance of a leveling of both overweight and obesity in some developed countries. Of greater awareness is the concern that the largest increase in overfat is due to both increases in the prevalence of abdominal overfat and the increasing average size of WCs, as abdominal overfat is the most serious form of the overfat pandemic. Bringing these factors into evidence could help lead to action in reducing the overfat pandemic.

Continued debate on how best to measure obesity and body fat may miss an important clinical point. That being, that most clinicians in the course of their normal in-office assessments usually know if the patient in front of them has too much body fat. Clinicians should not have to wait for the latest consensus or for a patients’ fat mass to reach a pre-established cutoff in order for them to implement appropriate lifestyle strategies to reduce excess body fat in their patients.

In the future, an accurate evaluation of the prevalence of overfat will need to include a consistent and accurate measurement of BFP across populations, as well as a general consensus on cutoffs along with factors associated with fat distribution, and the relationships with health risks. These evaluations of overfat should be based on epidemiologic studies with long-term follow-up and information on overfat-related comorbidities and mortality. However, this should not be done at the expense of population-wide effective remedies to reduce the incidence of the overfat pandemic and to prevent its growth by addressing the problem as it emerges in children.

## Conclusion

The estimate of overfat in the world’s 30 top developed nations is substantially higher than the prevalence of overweight and obese adults and children worldwide and stresses the seriousness of the overfat pandemic. Regardless of BMI values, overfat individuals have excess body fat, a high degree of cardiometabolic dysregulation that can promote disease risk factors and chronic disease, increased morbidity and mortality, reduced quality of life, and pose a rising economic burden. As an unfulfilled public health action, it is crucial to clinically identify individuals who are overfat in order to implement successful treatment and prevention strategies.

## Author Contributions

PM—originator of ideas, data collection, analysis, critical thinking, writing of the manuscript. IR-D—contributed to ideas, data analysis, figure and table writing, critical thinking, contributed to the writing. PL—contributed to ideas, critical thinking, writing of the manuscript.

## Conflict of Interest Statement

The authors declare that the research was conducted in the absence of any commercial or financial relationships that could be construed as a potential conflict of interest. PM is an independent clinical consultant, writes articles and books that include the topics presented herein, and has a business website pertaining to health and fitness (www.philmaffetone.com). IR-D works for a health and fitness company. PL is an independent consultant, writes articles and books, and has a website pertaining to performance, health, and longevity (www.plewsandprof.com).
